# The difference between concealment and blinding in clinical trials and why both are important. A reply to Garg and Mickenautsch, BMC Medical Research Methodology (2022) 22:17

**DOI:** 10.1186/s12874-023-02074-5

**Published:** 2023-11-21

**Authors:** Patrick Lyden, Joseph Broderick, James Grotta, Thomas Kwiatkowski, Steven Levine, Michael Frankel, E. Clarke Haley, Barbara Tilley

**Affiliations:** 1 Department of Physiology and Neuroscience, Zilkha Neurogenetic Institute of the Keck School of Medicine at USC, 1501 San Pablo Street, ZNI Room 245, Los Angeles, CA 90033 USA; 2 University of Cincinnati Gardner Neuroscience Institute, Cincinnati, OH USA; 3 Memorial Hermann Hospital, Texas Medical Center, Houston, TX USA; 4 Donald and Barbara Zucker School of Medicine at Hofstra/Northwell, Hempstead, NY USA; 5 State University of New York Downstate Health Sciences University, Brooklyn, NY USA; 6 Emory University School of Medicine, Marcus Stroke & Neuroscience Center at Grady Memorial Hospital, Atlanta, GA USA; 7 University of Virginia, Charlottesville, VA USA; 8 Department of Biostatistics and Bioinformatics, University of Texas Health Science Center, School of Public Health at Houston, Houston, TX USA

To the Editor,

We have been made aware of a paper in your journal [1] that purports to identify a risk of ‘selection bias’ in the two studies that comprised the NINDS rt-PA for Acute Ischemic Stroke Study [2], referred to from here on as the Studies. Upon careful reading, we find that the authors re-state some factual errors and misconceptions about the Studies. They also raise a new accusation that somehow patients with a more favorable prognosis were selectively directed into the rt-PA treated arm, thus biasing the trial toward a better outcome in the treated arm. In making this claim, the authors conflate two very different aspects of rigorous clinical trial design: treatment concealment versus blinding. We wish to untangle this confusion.

The authors first state there were 22 out of 624 (3.5%) cases that received incorrect treatment, of whom 21 received rt-PA instead of placebo. The Studies were conducted prior to the advent of the Internet or digital, web-based randomization systems: we used a manual randomization system of sequentially numbered envelopes. Vial numbers in the envelopes were assigned based on a stratified randomized block design and did not identify treatment group. To randomize, the investigator opened the next envelope in sequence to obtain a code number corresponding to a drug vial number. Although state of the art at the time, the system was complex and human errors occurred. There were 13 patients (2.1%) for whom the investigator chose the wrong vial ID number and of these, 11 (1.8%) received placebo but should have received rt-PA; two such patients received placebo and should have, so using the incorrect vial did not change treatment. These issues were all disclosed to the FDA in the final trial report. During review of the application for approval, an independent FDA reviewer commented that since the patients receiving placebo rather than rt-PA had better outcomes than the other placebo patients, “…*this error in the randomization process appears not to have contributed any bias to overestimating the treatment effect*.” (Full report is available at Clinical Review II for PLA 96–0350 (fda.gov)) In one patient, although the correct vial ID number was used, the coordinating center had packaged the wrong drug, and the patient received placebo but should have received rt-PA. In 18 (2.9%) patients, drug vials were chosen from the wrong treatment stratum, in part due to delays in drug re-supply. Of these 18 mis-randomizations, 11 ended up getting the wrong drug assignment: 10 (1.6%) received placebo who should have received active drug and one received rt-PA who should have been given placebo. Again, after considering the outcomes in these patients, the independent FDA reviewer concluded “…*the errors do not seem to have altered the overall outcome of the studies*.”

Next, the authors describe the well-known imbalance in mild stroke patients that favored the rt-PA treated group. This imbalance was adjusted for in the original analysis, and upon extensive, independent re-analysis, no impact on the results was found [3]. Then the authors go one step further and attempt to link the mis-randomized issue to the mild-NIHSS imbalance issue by noting that we opened the unblinding envelopes in 16 (2.6%) patients, 8 (1.3%) for patient bleeding, and 8 (1.3%) for unstated reasons. The authors mischaracterized these opened envelopes as “failed concealment” rather than unblinding.

The distinction between concealment and blinding is critically important. Concealment is used to reduce selection bias by assuring that the investigator who selects the subject and assigns treatment has no knowledge of the treatment about to be given. In contrast, blinding is used to reduce performance bias by assuring that all outcome assessments are done by an investigator without knowledge of the treatment group to avoid preferentially rating one group or the other as better. A failure of concealment occurs before treatment, while a failure of blinding occurs after treatment.

In no case was a treatment revealed (unblinded) prior to treatment. Unblinding occurred only after an adverse event or some other intercurrent clinical event. Bleeding is one such clinical event, and as a result, more unblinding occurred in the patients after rt-PA treatment, compared to placebo. It is crucial to note that there was not one single incident of failed concealment in the Studies, and no possibility of selection bias. Stated another way, the alleged ‘randomization subversion’ the authors assert was physically impossible.

The authors allude to the characteristic foaming of the drug and gum bleeding as further threats to concealment. First, the placebo drug clearly foamed the same as the active drug. In addition, gum bleeding could only occur AFTER treatment, and thus was not in any way a threat to concealment. Nothing enabled the investigator randomizing the patient to knowingly allocate patients with lower NIHSS scores to one group over another.

After a re-analysis of the data that the authors label ‘sensitivity analysis’ purporting to correct for selection bias, they present their ‘revised effect size’ in their Table 4. We note that all of the odds ratios still favor treatment, while some of the revised confidence intervals contain 1.0. The authors ignored, however, recalculating the primary outcome analysis of the trial, which used a global odds ratio. We wonder why? They offer their opinion that the global odds ratio has no clinical meaning, yet it was accepted by the FDA as representing the ability to statistically determine a “consistent and persuasive treatment effect” using multiple outcome measures.

The Studies’ results have been confirmed in further randomized, controlled trials [4, 5], and in the daily practice of vascular neurology where all of us witness the benefit of thrombolysis every day [6–8]. We write now to flatly deny that somehow treatment assignment was manipulated. Concealment failure was not only antithetical to us, it was physically impossible.

Most importantly, we write to firmly refute any suggestion that acute stroke patients should not be treated with thrombolysis. To withhold proven therapy from appropriate patients is unjustified. There is no equipoise in the vascular neurology community nor is there enthusiasm for a placebo-controlled rt-PA trial within 3 h of stroke onset. The benefit of thrombolytic therapy for acute ischemic stroke is supported by a considerable published literature and is considered standard of care around the world. We urge all readers to review the literature personally and carefully judge the results of the many large, rigorous, well-designed trials—including ours—that establish the considerable benefit of intravenous thrombolysis for acute ischemic stroke.

## Data Availability

All data from the original trial is available from the NINDS.

## Abbreviations

NINDS: National Institute of Neurological Disorders and Stroke
rt-PA: Recombinant tissue type plasminogen activator
FDA: Food and Drug Administration
